# Maternal Deaths due to Obstetric Haemorrhage in Dodoma Regional Referral Hospital, Tanzania

**DOI:** 10.1155/2020/8854498

**Published:** 2020-11-12

**Authors:** Mzee M. Nassoro, Enid Chiwanga, Athanase Lilungulu, Deogratius Bintabara

**Affiliations:** ^1^Department of Obstetrics and Gynaecology, Dodoma Regional Referral Hospital, Dodoma, Tanzania; ^2^Department of Obstetrics and Gynaecology, University of Dodoma, College of Health Sciences, Dodoma, Tanzania; ^3^Department of Public Health, College of Health Sciences, University of Dodoma, Dodoma, Tanzania

## Abstract

**Background:**

Despite the availability of comprehensive emergency obstetric care at Dodoma Regional Referral Hospital, deaths due to obstetric haemorrhage are still high. This study was carried out to analyse the circumstances that had caused these deaths.

**Methods:**

A retrospective review of all files of women who had died of obstetric haemorrhage from January 2018 to December 2019 was made.

**Results:**

A total of 18,296 women gave birth at DRRH; out of these, 61 died of pregnancy-related complications of the deceased while 23 (38%) died of haemorrhage, with many of them 10 (44%) between the age of 30 and 34. Many were grand multiparous women 8 (35%) and almost half of them (11 (48%)) had stayed at DRRH for less than 24 hours. More than half (12 (52%)) had delivered by caesarean section followed by laparotomy due to ruptured uterus (8 (35%)). The leading contributing factors to the deaths of these women were late referral (6 (26%)), delays in managing postpartum haemorrhage due to uterine atony (4 (17%)), inadequate preparations in patients with the possibility of developing PPH (4 (17%)), and delay in performing caesarean section (3 (13%)).

**Conclusion:**

Maternal mortality due to obstetric haemorrhage is high at Dodoma Regional Referral Hospital where more than one-third of women died between 2018 and 2019. Almost all of these deaths were avoidable. The leading contributing factors were late referral from other health facilities, inadequate skills in managing PPH due to uterine atony, delays in performing caesarean section at DRRH, and inadequate preparation for managing PPH in patients with abruptio placentae and IUFD which are risk factors for the condition. There is a need of conducting supportive supervision, mentorship, and other modes of teaching programmes on the management of obstetric haemorrhage to health care workers of referring facilities as well as those at DRRH. Monitoring of labour by using partograph and identifying pregnant women at risk should also be emphasized in order to avoid uterine rupture.

## 1. Introduction

Deaths from complications of pregnancy and childbirth are still high with WHO recording 295,000 maternal deaths globally in 2017 [1]. Obviously this trend is not much different from that noticed during the assessment of achievements of the MDGs in 2015. At that time, global maternal mortality ratio, though decreased to 196 per 100,000 from 282 per 100,000 of 1990, did not reach the agreed target of 75% reduction [2].

Disproportionally, about 94% of these deaths occur in low-income countries. In year 2017, sub-Saharan Africa alone contributed to about two-thirds (196,000) of all global maternal deaths with ten countries including Tanzania, contributing to 59% of these deaths [1].

Causes of maternal deaths are classically divided into direct and indirect categories. Direct causes are those which are directly associated with the pregnancy itself or its management while indirect causes are those conditions which are worsened by pregnancy. Direct causes are haemorrhage, eclampsia, and complications of obstructed labour, complications of unsafe abortion, sepsis, thromboembolism, and anaesthetic complications. The major indirect causes are malaria, anaemia, and HIV/AIDS. Globally, women die mostly due to direct obstetric causes. Between years 2003 and 2009, such causes contributed to 73% of all maternal deaths with 27% of women dying of haemorrhage [3].

In a literature review relating to factors associated with persistent maternal mortality caused by postpartum haemorrhage in sub-Saharan Africa in 2010, it was found that of 800 women who died every day due to birth complications 440 were from this region and PPH was the main cause [4]. Moreover, in their study, Luis Alvarez et al. found that 34% of maternal deaths in Africa were due to haemorrhage [5]. In East Africa, a study conducted in the central region of Kenya showed that haemorrhage contributed to 40% of all causes of maternal deaths and the majority of these women died within 24 hours after admission [6].

In Tanzania, the Demographic and Health Survey (DHS) 2015 and 2016 established that the country's maternal mortality ratio was 556 per 100,000 live births but did not specifically identify the causes of death [7]. However, a recent study of health facility data collected by the Ministry of Health, Gender Development, Elderly and Children from All Regions in Mainland Tanzania, showed that haemorrhage was the leading cause of maternal mortality in Tanzania [8]. Other studies on causes of maternal mortality occurring at the national and regional referral hospital levels also showed that haemorrhage was the leading cause [9].

In Dodoma Regional Referral Hospital, haemorrhage has also been identified as one of the leading causes of maternal deaths, which, if could be dealt with properly, can significantly reduce maternal mortality [10]. We have undertaken this study to identify circumstances surrounding these deaths in order to advice and mitigate the situation and improve maternal outcome in women developing obstetric haemorrhage.

## 2. Methodology

### 2.1. Study Design

This is a retrospective case review of maternal deaths that occurred in Dodoma Regional Referral Hospital in 2018 and 2019 due to haemorrhage.

### 2.2. Study Setting

The study setting was at the Maternity block in Dodoma Regional Referral Hospital, Dodoma city. This is the oldest public hospital in Dodoma region serving as a referral centre for all councils in Dodoma with population of 2.2 million people. It also serves patients from neighbouring districts of neighbouring regions (Gairo, Morogoro), (Kiteto, Manyara), and (Manyoni, Singida).

The Maternity Block is a 2-storey building with bed capacity of 130. Thirty to 40 women deliver per day with a caesarean section rate of 22%. Apart from those women coming for delivery, others are admitted for pregnancy complications, gynaecological problems, neonatal problems, and prematurity.

On the ground floor are the registration window, consultation rooms, a small laboratory, pharmacy, gynaecological theatre, and ward. The first floor consists of the labour ward, 10 delivery cubicles with a newborn resuscitation room, obstetric theatre, and a ward for women who underwent caesarean section and those with postpartum complications such as puerperal sepsis, anaemia, etc. There is a special room for patients with hypertensive disorders of pregnancy from gestation age of 28 weeks and above on this floor too. The second floor accommodates antenatal and postnatal patients in separate halls. Still, on the same floor, there is a wing for sick neonates and a premature unit.

The workforce in the department of obstetrics and gynaecology consists of four specialists and three registrars. There are also an average of 10 intern doctors who rotate in the department every three months. As part of their program, residents studying obstetrics and gynaecology from the University of Dodoma spend one year of their clinical studies in the department and there were five of them during this study.

The nursing staff consists of 53 registered nurses and nurse midwives working in three shifts arrangement; each shift is managed by 2 to 4 nurses. Total deliveries range from 30 to 40 in 24 hours with a caesarean section rate of 22%.

### 2.3. Data Collection

All maternal death files from January 1, 2018 to December 31, 2019 were retrieved from the maternal and perinatal death surveillance and response focal person. The author went through every file to identify those in which haemorrhage was the cause. Although ruptured uterus is considered as a cause of death on its own, in this study it has been included as a cause of death due to haemorrhage. Of the reviewed files, 24 were identified as of those who experienced haemorrhage and died; however, one of the deceased was managed for a ruptured uterus and died 5 days later due to sepsis. This file was excluded and only 23 were analysed.

The information collected included age, parity, mode of admission, duration of hospital stay, mode of delivery, and contributing factor to the death due to haemorrhage.

### 2.4. Data Analysis

The author checked each variable manually, tallied its occurrence on a sheet of paper, made summations, and calculated frequencies. The data collected was then entered into the tables and analysis was made.

### 2.5. Ethical Clearance

This review was approved by the Dodoma Regional Referral Hospital research committee. Anonymity of the deceased and the health care providers was maintained as required by guidelines when discussing maternal deaths.

## 3. Results

During the study period, 18,296 women gave birth at DRRH (8,722 in 2018 and 9,574 in 2019). There was a total of 61 maternal deaths in the same period, 34 in 2018 and 27 in 2019. Eleven (32%) and 12 (44%) maternal deaths were due to haemorrhage in 2018 and 2019, respectively (Table 1).

The youngest deceased was 21 while the eldest was 40 years. Many maternal deaths occurred to women between 30 and 34 years (44%) with most of them being multipara 8 (35%). There was almost equal number of those who were admitted as referrals and those who came direct from home. The duration of hospital stay ranged from 1 hour to 5 days, with most of the women 11 (48%) dying within 24 hours of admission (Table 2).

More than half of the deceased delivered by caesarean section 12 (52%) and 8 (35%) delivered by laparotomy due to uterine rupture (Table 3).

Delayed referral from another facility was the leading contributing factor to the deaths 6 (26%) followed by delay in managing uterine atony 4 (17%) and inadequate preparation in patients with possibility of developing postpartum haemorrhage 4 (17%) (Table 4). Others were delay in performing caesarean section at Dodoma Regional Referral Hospital 3 (13%), inadequate skills in repairing deep vaginal tears and lacerations 2 (9%), delay in recognising ruptured uterus 2 (9%), inadequate skills in performing caesarean section 1 (4%), and a complicated caesarean section 1 (4%) (Table 4).

## 4. Discussion

Bleeding, either before or after birth, is a major cause of maternal deaths. Most of these deaths are avoidable at different levels of health care system if there are competent staff, adequate medicines, and medical supplies. A regional referral hospital is at the level where skills are supposed to be abundant as well as medicines and medical supplies to avert maternal deaths due to haemorrhage. This study, which was conducted in one of the prominent regional referral hospitals in central Tanzania, provides very important information regarding contributing factors to haemorrhage as a cause of maternal deaths.

Obstetric haemorrhage contributed to 38% of all maternal deaths in the study period. Similar findings were recorded in a retrospective analysis of causes of health facility maternal deaths in Ogun state, Nigeria, and central regions of Kenya where haemorrhage was the leading cause accounting for 40% and 43.3%, respectively [6, 11]. A study conducted at Shinyanga Regional Referral Hospital, Tanzania, also correlates to the above findings that haemorrhage was the leading cause of maternal deaths by 50% [12]. In another study, also conducted in Tanzania, Bwana et al. found that haemorrhage was second to eclampsia in causing deaths at 24.6% [13]. However, contrary to our study, this study did not classify uterine rupture as one of the causes of haemorrhage. Reasons for haemorrhage being the leading cause of maternal deaths at DRRH will be discussed further in the following paragraphs.

DRRH is the only public health facility in the region that receives most of referred complicated obstetric patients. Being located in the city centre, the hospital is easily accessible and women can be admitted directly from home without referral. In this study, the number of women who were referred was almost as equal as that of those admitted from home, i.e., 52% and 48%, respectively (Table 2). This result differs from that of other studies of maternal mortality undertaken in referral hospitals. In one such study in South Africa, it was found that most of the women (89%) who had died were referred from other health facilities [14]. This difference may be attributable to better referral systems in such countries as South Africa compared to Tanzania.

In order to avert maternal deaths, prompt intervention is required when an obstetric emergency occurs. It has been estimated that average time between the onset of such an emergency and death is twelve hours without intervention. Of all obstetric emergencies, haemorrhages, especially postpartum haemorrhage, can kill a woman very fast. Maine estimated that antepartum haemorrhage can be fatal within 12 hours, postpartum haemorrhage within 2 hours, and ruptured uterus within 1 day [15, 16]. In our study, 70% of women died within 48 hours of admission, most of them (48%) in the first 24 hours (Table 2). The range of hospital stay was between one hour and five days. While reviewing the records, we realised that those who stayed for a short time before death were referred late and had ruptured uteruses (Table 3). This finding further emphasizes on early recognition of women at risk of rupturing uterus and those with poor progress of labour in facilities which cannot perform caesarean section in order to facilitate timely referrals. The problem of referring women already with ruptured uteruses is also frequently seen in other low- and mid-income countries such as India where a study conducted in Karnataka Institute of Medical Sciences showed that 72% of referred women had ruptured uteruses [17]. However, 5 (21%) women in our study had ruptured uteruses while already at DRRH. Of these, 3 were already scheduled for caesarean section but there were delays in performing the procedure. The delays were caused by inappropriate triaging of the women needing caesarean section in the face of inadequate theatre space. Such delays and their results call for reviewing or developing a triage protocol and making use of the available general theatre when there are so many pregnant women needing immediate surgical intervention. The other 2 (9%) patients had ruptured uteruses right in the DRRH labour ward without knowledge of either the doctor or nurse on duty. Despite being small, this number represents lost lives and possible adverse consequences to the families of the deceased. Maternal deaths under such circumstances may just be a tip of the iceberg hinting to inadequate monitoring of the women while in labour due to either inadequate skills, shortage, or attitude of staff. This problem needs to be addressed in all these angles by building staff capacity in skills and attitude and also in modifying job allocation such that every nurse on duty is assigned particular patients to take care of and write a report for anything that goes wrong. This will enhance the sense of responsibility and accountability for individual health care workers.

Two patients died due to haemorrhage after caesarean section. One was due to inadequate skills of the doctor who had not sutured the uterine incision at one of the lateral angles. This fault was discovered during a second operation which was performed because the patient was still bleeding significantly after the procedure. Unfortunately, this patient died of irreversible shock. The other patient died because of uncontrolled bleeding during caesarean section. This patient was HIV positive with a previous caesarean section scar. The surgeon also noted that there were so many adhesions and it was difficult to control bleeding. This shows how caesarean section which is meant to save either the mother or the foetus can actually become the cause of death. In a recent study on maternal mortality in the Netherlands, it was found that maternal mortality following caesarean section was three times higher compared to vaginal birth, and another study in Sierra Leone found that the cause of death was mostly haemorrhage (73%) [18, 19]. Risks of dying due to caesarean section can be reduced by encouraging the health care providers to analyse poor progress of labour and make use of augmentation where applicable and also encourage vaginal birth after caesarean section with all necessary precautions. Skills in part of those performing the procedure are of paramount importance and haemorrhage should be dealt with immediately if it complicates the procedure. In some situations, caesarean hysterectomy should be performed in order to save the woman's life even if she is of low parity.

In the three-delay model depicted by Thaddeus and Maine, the third delay involves the health facility level [20]. This can be in terms of delayed referral from one health facility to another, delayed intervention at a health facility, or inadequate skills of a health care provider. In this study, third delay contributed to all deaths. Delayed referral from lower health facilities to DRRH contributed most (26%). Similar findings were noted in a study conducted in Mali and Senegal where delayed referral in patients with PPH caused a 50% case fatality rate [21]. The reasons for this delay in our study were not well documented but could be due to inadequate skills of the providers at these levels in recognising the need to refer the patients or availability of transport for referral. This calls for strengthening the capacity of health care workers in early recognition of danger indicators as well as equipping the lower facilities with means of immediate transport for referral patients. The health care workers, both in peripheral and referral hospitals like DRRH, should be equipped with skills in recognising risk factors for development of uterine rupture and in monitoring labour using a partograph. Many women in this study could have been saved if health care workers had such skills. The newly constructed Comprehensive Emergency Obstetrics and Newborn care facilities in all districts surrounding DRRH need to be complemented with adequate equipment, instruments, medicines, medical supplies, and skilled human resources. With the current flow and reasons for referral to DRRH, there seems to be gaps needing to be addressed for these facilities to function fully and reduce maternal mortality due to haemorrhage and other causes in Dodoma region.

Uterine atony is a well-known leading cause of maternal deaths due to haemorrhage. A multicentre study in South America found that 75% of women who had died of PPH had uterine atony [22]. At DRRH, 17% of patients died because of delays in managing PPH due to uterine atony. All of these patients had had uterotonics administered for active management of the third stage of labour; however, it did not work. In such situation, a health care worker is required to employ other means of management of PPH such as aortic artery compression, bimanual compression of the uterus, intrauterine balloon tamponade, and rigorous intravenous fluid and surgical intervention if everything fails. Swift decision making is very important in postpartum haemorrhage. Delays in the management of these patients could have been due to inadequate skills of providers especially the intern doctors and nurses who are the first in seeing patients with such problems.

Abruptio placentae and intrauterine fetal death carry the risk of developing consumptive coagulopathy [23]. When attending such patients in labour, the health care worker should keep this in mind and perform coagulation profile like bed side clotting time, activated partial thromboplastin time, and prothrombin time. Moreover, adequate crystalloid solutions, blood and fresh frozen plasma should be available for maintaining circulation and clotting factors. In this study, 4 patients (17%) died of postpartum haemorrhage because of coagulopathy secondary to abruptio placentae and intrauterine fetal death. There was no workup to investigate these patient's coagulation profile, and when they developed PPH, there were no adequate interventions to save their lives. Health care workers should, at all times, be prepared for PPH when attending these women.

Two (9%) patients in this study died because of faulty repair of deep lateral vaginal tears leading to continuing PPH. These deaths could have been avoided if these deep vaginal tears had been repaired adequately and on time. Furthermore, episiotomies performed earlier on could have abated the tears hence haemorrhage and deaths of these women. Contrary to our study, in a study done in Nigeria, such tears were found to have occurred in women who had delivered outside health facility and caused very low mortality [24].

This study has highlighted gaps in general management of patients and those with obstetric haemorrhage at DRRH. Like in other studies, it has also shown great contribution of late referral to the deaths of women with haemorrhage [17, 21, 22]. Furthermore, delays in interventions and inadequate skills of providers had also contributed to the deaths. The skill problem is especially with lower clinical cadres such as intern doctors and nurses. This calls for duties to be arranged in such a way that every shift has an experienced provider.

There were limitations to this studies due to its nature of being retrospective. The most challenging task was to retrieve the information from the files which are, actually, a collection of improperly arranged papers. Secondly, management charts were either not completely filled or were missing from many files.

The Tanzanian Government advocates facility delivery in order to prevent maternal mortality and morbidity. The findings in this study have highlighted gaps in intervention when dealing with obstetric haemorrhage, which is a global leading cause of maternal deaths.

## 5. Conclusion

DRRH has high mortality due to obstetric haemorrhage. More than a third of women who died in 2018 and 2019 had haemorrhage as the cause. Most of them (44%) were between 30 and 34 years of age and many (35%) were grand multipara. Almost a half (48%) of deceased women had stayed at DRRH for less than 24 hours. More than half (52%) delivered by caesarean section with many (26%) having been referred late. Inadequate skills of some health care providers in managing obstetric haemorrhage and late intervention at DRRH had also contributed to these deaths. There were deaths due to uterine rupture that had occurred at DRRH. By going through the file notes, we have found that almost all of these deaths were avoidable if quick and appropriate measures were taken.

The findings in this study call for improving the quality of care of facilities referring women to DRRH by building capacity of health care providers through mentorship and other forms of training to enable them manage obstetric haemorrhage and provide early referral when needed. It is of great importance to mentor and supervise the lower cadre health care providers (intern doctors and nurses) at DRRH so that they can monitor labour according to the standards and intervene immediately when there is deviation. All shifts should have at least one health care provider who is very conversant with performing caesarean section and manage obstetric haemorrhage. There needs to be another study which will show prevention of postpartum haemorrhage by performing active management of the third stage of labour and blood transfusion in all women who develop postpartum haemorrhage at DRRH.

## Figures and Tables

**Table 1:** All maternal deaths in 2018–2019 (*N* = 61).

2018			2019	
Characteristic	*N*	%	*N*	%
Maternal deaths due to haemorrhage	11	33	12	44
Maternal deaths due to other causes	23	68	15	56
Total maternal deaths	**34**		**27**	

Twenty-three women out of 61 died of obstetric haemorrhage.

**Table 2:** Demographic characteristics, mode of admission, and duration of hospital stay of the deceased due to haemorrhage (*N* = 23).

Characteristic	*N*	%
Age		
20–24	3	13
25–29	4	17
30–34	10	44
35 and above	6	26

Parity		
1	2	9
2	6	26
3	2	9
4	4	17
5 and above	8	35
Unknown	1	4

Mode of admission		
Home	11	48
Referral from another facility	12	52
Unknown	1	4

Duration of hospital stay		
<24 hours	11	48
24 ± 48 hours	5	22
48 ± 72 hours	2	9
72 + and above	4	17
Unknown	1	4

Most the women were between 30 and 34 years and grand multiparous and almost half of them stayed at DRRH for less than 24 hours.

**Table 3:** Mode of delivery (*N* = 23).

Characteristic	*N*	%
Spontaneous vertex delivery	3	13
Caesarean section	12	52
Laparotomy for ruptured uterus	8	35

More than half of the women delivered by caesarean section.

**Table 4:** Contributing factors to the deaths (*N* = 23).

Characteristic	*N*	%
Delayed referral	6	26
Delay in performing caesarean section at DRRH	3	13
Delays in managing uterine atony at DRRH	4	17
Inadequate skills in repairing deep vaginal tears and lacerations at DRRH	2	9
Delay in recognising ruptured uterus at DRRH	2	9
Inadequate skills in performing caesarean section at DRRH	1	4
Inadequate preparations in patients with the possibility of developing PPH (abruptio placentae, IUFD) at DRRH	4	17
A complicated caesarean section in a patient with AIDS and a lot of adhesions	1	4

Delayed referral, delay in managing uterine atony, inadequate management of IUFD and abruptio placentae, and delays in performing caesarean section at DRRH contributed to most of these deaths.

## Data Availability

The data used to conduct this study was extracted from the hospital files of the deceased and are included within the article. The files are kept under the custodianship of the Maternal and Perinatal Death Surveillance and Response focal person.
